# Vulnerability as a Function of Individual and Group Resources in Cumulative Risk Assessment

**DOI:** 10.1289/ehp.9332

**Published:** 2007-01-24

**Authors:** Peter L. deFur, Gary W. Evans, Elaine A. Cohen Hubal, Amy D. Kyle, Rachel A. Morello-Frosch, David R. Williams

**Affiliations:** 1 Virginia Commonwealth University, Richmond, Virginia, USA; 2 Cornell University, Ithaca, New York, USA; 3 U.S. Environmental Protection Agency, National Center for Computational Toxicology, Research Triangle Park, North Carolina, USA; 4 University of California Berkeley, Berkeley, California, USA; 5 Brown University, Providence, Rhode Island, USA; 6 Harvard School of Public Health, Boston, Massachusetts, USA

**Keywords:** communities, cumulative risk, environmental justice, public health, vulnerability

## Abstract

**Background:**

The field of risk assessment has focused on protecting the health of individual people or populations of wildlife from single risks, mostly from chemical exposure. The U.S. Environmental Protection Agency recently began to address multiple risks to communities in the “Framework for Cumulative Risk Assessment” [EPA/630/P02/001F. Washington DC:Risk Assessment Forum, U.S. Environmental Protection Agency (2003)].

Simultaneously, several reports concluded that some individuals and groups are more vulnerable to environmental risks than the general population. However, vulnerability has received little specific attention in the risk assessment literature.

**Objective:**

Our objective is to examine the issue of vulnerability in cumulative risk assessment and present a conceptual framework rather than a comprehensive review of the literature. In this article we consider similarities between ecologic and human communities and the factors that make communities vulnerable to environmental risks.

**Discussion:**

The literature provides substantial evidence on single environmental factors and simple conditions that increase vulnerability or reduce resilience for humans and ecologic systems. This observation is especially true for individual people and populations of wildlife. Little research directly addresses the topic of vulnerability in cumulative risk situations, especially at the community level. The community level of organization has not been adequately considered as an end point in either human or ecologic risk assessment. Furthermore, current information on human risk does not completely explain the level of response in cumulative risk conditions. Ecologic risk situations are similarly more complex and unpredictable for cases of cumulative risk.

**Conclusions:**

Psychosocial conditions and responses are the principal missing element for humans. We propose a model for including psychologic and social factors as an integral component of cumulative risk assessment.

Risk assessment methods are widely used to assess environmental problems, but current methods are not designed to address risks of cumulative exposures to environmental stressors. To begin to remedy this, the U.S. Environmental Protection Agency developed the “Framework for Cumulative Risk Assessment” (referred to in this article as “Framework”; U.S. EPA 2003) that sets out general practices for cumulative risk assessment and called attention to critical gaps. One critical gap was the difference in vulnerability among individuals, communities, and populations. Any underlying vulnerability could increase the health impact of exposure to environmental agents. The Framework also observed that risk assessment methods consider risks to individuals or populations but fail to evaluate risks to communities and recommended determining how to include and measure vulnerability of individuals or groups. This article is one of five articles in a mini-monograph on cumulative risk (Callahan and Sexton 2007) and explores ways in which to address certain elements of vulnerability in groups such ecologic or human communities.

Kasperson et al. (1995) define vulnerability as “The propensity of social or ecological systems to suffer harm from external stresses and perturbations.” The four properties of vulnerability used in the Framework (U.S. EPA 2003), taken from [National Environmental Justice Advisory Committee (NEJAC) 2004] are susceptibility, exposure, preparedness, and responsiveness. Most important, vulnerability is how individuals or groups of individuals or organisms respond to and recover from stressors inadequately or not as well as the average. We focus here on nontoxicologic vulnerabilities, especially psychosocial stress and responses, community structure and function, and population assessment and response.

In this article we discuss the factors that affect how a person, an animal, an ecologic population, or community might be more (or less) vulnerable because of their capacities and resources, coping mechanisms, supports, and size and complexity of the group. We consider both human communities, which include the cultural and social elements in addition to the obvious food, transportation, medical systems, and so forth, and ecologic communities, which include the plants, animals, and microbes in a habitat or habitats. Issues of susceptibility, properties of the individual or group, are integrated into the discussion on preparedness and responsiveness; differential exposure is considered in another article in this mini-monograph (Sexton and Hattis 2007). Assessment of multiple stressors is examined by Menzie et al. (2007), and Ryan et al. (2007) discuss using biomarkers in cumulative risk assessment.

In this article, we emphasize differential preparedness and the ability of an animal or group to recover under cumulative risk situations. There is a distinction between the two aspects of vulnerability. Differential preparedness is an underlying mechanism or process representing the coping mechanisms and resources that an animal or group displays in advance of the stress condition (Kasperson et al. 1995). We consider the ability to recover to reflect traits that allow the organism, individual, or group to heal from or compensate for the effects of exposure to environmental agents or stressors. Resilience is similar to recovery in responding better than the average. In ecologic terms, resilience is the ability of a population to continue on in time and space (Holling 1973); the difference between stability and resilience is returning to the same state (stability) versus maintaining relationships among variables (resilence). A resilient individual or group is subject to harm from stressors but is able to overcome stress conditions.

The present analysis adds psychosocial stress to the usual list of stressors that are evaluated in risk assessments: chemical, physical and biological stressors. Psychosocial stress refers to everyday chronic stressful experiences related to social environments in families, the household, the workplace, neighborhoods, schools, etc. Chronic stress is the cumulative load of minor or major day-to-day stressors that can have long-term health consequences and potentially lead to immune dysfunction (Geronimus 1992, 2000; McEwen 1998). This type of stress is not restricted to humans but can occur in ecologic systems with the imposition of such factors as increased predators and habitat degradation or crowding.

The purpose of this article is to add vulnerability to a framework for cumulative risk assessment. A comprehensive review of the literature and exploration of all the issues related to cumulative risk, ecologic and human vulnerability, and other issues is well beyond the scope of this article. We propose a conceptual model for how vulnerability factors may be incorporated into a cumulative risk assessment. We discuss additional information that would need to be measured, collected, and tracked to ensure that the full range of stressors and mediating influences are considered in a cumulative risk assessment. Finally, we present consideration of research needed to better determine the relationship between psychosocial conditions and environmental health.

## Vulnerability Factors Relevant to Cumulative Risk

Cumulative risk assessment builds on traditional risk assessment methods, which are centered on a source–exposure–response paradigm. Indeed, the National Research Council (NRC 1983) emphasized such a framework with four components: exposure assessment, hazard assessment, dose–response estimation, and risk characterization. In this article we incorporate psychosocial factors into a cumulative risk assessment and recognize that vulnerability can be relevant to both individuals and communities. Health outcomes are predicted by the relationships among measures of environmental conditions (stressors), receptor characteristics (measures of potential vulnerability), and receptor resources (abilities to respond or recover). For a human community, relevant environmental conditions may include ambient environmental quality, neighborhood safety, and type of housing. The community may be characterized by racial/ ethnic composition, socioeconomic composition, and health status. Resources available to the community may include health care, educational and employment opportunities, commercial establishments, and transportation. An ecologic community may be characterized by available resources, the number of species and their proportionate representation, genetic diversity, health status, and total number and mass of animals.

Table 1 lists vulnerability factors that are characteristic of both the environment–receptor interaction and the receptor–response function for humans and wildlife. The dynamic nature of this system is highlighted in the overlap of many vulnerability factors across the general categories. Socioeconomic status (SES), for example, is a social factor associated with the receptor as well as a resource associated with the social environment. Examples of more specific vulnerability factors are also presented.

Most of these vulnerability factors are expressed in terms that are most applicable to people, but many apply to ecologic systems. Habitats may be disturbed or intact, diminished or expanded, close to human activities or not, water limited or flooded, and so forth. The biological conditions such as health, disease, nutrition, genetic makeup, activity levels, and stress also apply directly to wildlife. Unfortunately, an encyclopedic comparison of human and ecologic factors in cumulative risk assessment is beyond the scope of this article. The social factors for wildlife are not the same as those for humans but are present and significant, at least for most terrestrial vertebrates such as birds, rodents, deer, other large mammals (terrestrial and marine), and rare and endangered animals.

### Differential exposure

Although differential exposures have been addressed by Sexton and Hattis (2006), it is important to note that disparities in environmental exposures probably play an important, albeit poorly understood, role in the origins and persistence of health disparities by race and SES, which can be augmented by vulnerabilities. A growing literature shows that exposures to environmental hazards often differ by race and SES, including estimates of proximity to emissions sources such as hazardous waste and large industrial facilities (Boer et al. 1997; Bullard 1983; Burke 1993; Commission for Racial Justice 1987; Hersh 1995; Mohai and Bryant 1992; Pastor et al. 2001; Pollock and Vittas 1995; Pulido et al. 1996; Sadd et al. 1999) exposure to specific substances such as pesticides and lead (Kraft and Scheberle 1995; Moses et al. 1993), exposures to outdoor air pollution and associated health risks (Gelobter 1992, 1993; Morello-Frosch et al. 2001) differences in regulatory enforcement (e.g., Superfund cleanups) (Hird 1993; Lavelle and Coyle 1992; Zimmerman 1993), proximity to Superfund sites (Baibergenova et al. 2003), and body burden measurements (Centers for Disease Control and Prevention 2003). The evidence suggests a pattern of disproportionate exposures to environmental risks among communities of color and the poor, with racial differences often persisting across economic strata.

### Psychosocial stress

Risk assessment methods to date have addressed chemical and biological stressors but have not addressed psychosocial stress. The direct effect of hazardous social and physical environments can combine with psychosocial stress. The resulting combination can further widen health disparities along racial and socioeconomic lines. The risk assessment framework implies that the emission or presence of an environmental agent must first lead to exposure and overcome the individual’s or communities’ defense systems to have an adverse effect. However, this model does not consider the possibility that the mere presence of the source of a stressor presents a psychologic reaction in individuals or communities, creating a psychosocial stress that can contribute to disease and may have physiologic elements such as elevated stress hormones.

The mechanism by which psychosocial stress increases individual and community vulnerability is not clear. Some studies suggest that psychosocial stress may alter the effects of toxic pollutant exposures such as environmental tobacco smoke (ETS). Psychosocial stresses may also result from exposures to other toxic chemicals and subsequent synergistic interactions (Whyatt et al. 2002). Stress may have a biological impact by amplifying differential vulnerability to the toxic effects of pollutants and by weakening the ability to recover from harmful exposures. Furthermore, stress alone may lead directly to illness, in turn rendering the individual more susceptible to toxic effects. Illness may also compromise the capacity to cope and recover from the adverse effects of environmental exposures (Rios et al. 1993). Finally, the literature suggests that both individual and community-level stressors can differentially moderate exposure–response relationships (Diez-Roux 1997, 1998, 2000; Rauh et al. 2004). Therefore, it is important to examine both levels of stressors to assess their impact on health outcomes that are both environmentally and socially mediated.

## Individual-Level Analysis of Human Vulnerability

Vulnerability in individuals can be classified according to characteristics of either the environment or the receptors (Tables 2 and 3). Little empirical research has been devoted to understanding the effects of vulnerability on cumulative risk. Thus, we draw largely upon work examining singular risk factors and vulnerability, assuming that characteristics affecting vulnerability to a singular risk factor likely will alter vulnerability to cumulative risks. We also survey research on resilience or protective factors that buffer adverse reactions to singular or cumulative risks because these resilience characteristics may also provide insights into the role of vulnerability factors in cumulative risk.

### Environmental characteristics

A wide range of investigations have demonstrated the negative effects of a variety of poor environmental conditions combined with chronic risks on health, as measured by physiologic functions, psychologic reactions, and mental health. Adult mental health, for example, is negatively affected by poor neighborhood quality and substandard housing (Kasl et al. 1982); lower quality neighborhoods coupled with social stressors (e.g., marital conflict) (Caspi et al. 1987); high residential density under multifamily dwelling conditions (Mitchell 1971), which is a condition also shown to affect children’s mental health (Evans et al. 2002); psychologic stress in addition to high smog (Evans et al. 1987); psychologic stress and residential crowding (Lepore et al. 1991). In addition, respiratory health symptoms from air pollutants on the job are greater among those also experiencing job stress (House et al. 1979). Physiologic stress (cardiovascular, neuroendocrine) responses to occupational noise exposure are elevated by higher task demands at work (Melamed et al. 2001; Welch 1979) among those reporting more job stress (Talbott et al. 1985) and for those with greater job dissatisfaction (Lercher et al. 1993).

Both children and adults exhibit greater physiologic reactivity (e.g., increase in blood pressure in response to an acute laboratory stressor such as mental arithmetic) and slower physiologic recovery (e.g., time to return to baseline for blood pressure) if they are also experiencing ongoing, background stressors in their daily life (Gee and Takeuchi 2004; Gump and Matthews 1999; Lepore et al. 1997). Family turmoil under conditions of residential crowding negatively affects children’s mental health and physiologic stress (Evans and Saegert 2000).

Environmental conditions may also be ameliorative and confer some resilience. An ongoing, consistent relationship with a caring and responsive adult significantly attenuates children’s adverse socioemotional and cognitive reactions to early childhood risk factors (Masten and Coatsworth 1998). Positive social conditions can protect against negative outcomes (Elder and Conger 2000; NRC 2002), and socially supportive relationships offer some modicum of protection for adverse psychologic and physical reactions to a variety of life stressors and ongoing life demands (Cohen and Wills 1985; House et al. 1988; Lepore 1997). Many of the individual-level vulnerability factors are summarized in Table 2, focusing on the social and community environment that warrant consideration in studies of individual-level vulnerability factors.

### Receptor characteristics—psychosocial dimension

The psychosocial situation of individuals can greatly affect their vulnerability. Vulnerability to cumulative risk exposure among primary school children is higher among those with negative emotionality (fearfulness, irritability, startle responses) (Lengua 2002). This point is consistent with a large body of literature indicating that young children with difficult temperament fare much worse in the face of risky environments than their counterparts with more positive temperament (i.e., easy going, better self-regulatory skills) (Masten and Coatsworth 1998; Repetti et al. 2002). Similarly, adults who have more negative affectivity (pervasive negative mood, anger) are also more vulnerable to harmful psychologic and physiologic consequences of stressors (Taylor 1999).

Resilience to stressors among children is enhanced by intelligence and positive temperament (sociability, easy going) (Masten and Coatsworth 1998). There is also evidence that children with better self-regulatory abilities, which appear to have both cognitive (e.g., attention allocation) and socioemotional components (e.g., impulse control, delay of gratification), are better able to cope with stress (Eisenberg et al. 1997; Mischel et al. 1989).

One of the most robust moderators of the negative impacts of risk factors among children and adults is a sense of control or belief in self-efficacy. Having the perception that one can regulate the degree of negative environmental circumstances one is facing has profound effects on both psychologic and physiologic health outcomes (Cohen et al. 1986; Glass and Singer 1972; Taylor et al. 1997). This pattern is also true in occupational situations (Karasek and Theorell 1990).

Among adults, optimism appears to offer protection against a wide range of physical and psychologically threatening conditions (Scheier and Bridges 1995). Optimists tend to cope with stressors either by engaging the demands or not disengaging by withdrawal or denial, two forms of maladaptive coping. Problem solving or accommodation appears to be a more effective coping strategy across a wide range of situations (Compas et al. 2001; Holahan et al. 1996; Lazarus and Folkman 1984). Early childhood positive temperament may well be a forerunner of optimism among adults.

Sex seems to influence vulnerability to psychologic stressors. Among children, boys prior to puberty are generally more vulnerable to a wide range of stressors than are girls, whereas after puberty, girls emerge as more vulnerable for depression and psychosomatic symptoms to stressors (Steinberg 2002). In adults, women tend to show less physiologic reactivity to stressors than men (Matthews and Stoney 1988). Some possible receptor factors that may influence individual vulnerability to cumulative risks are summarized in Table 3.

Although there are biological differences by sex that may affect vulnerability to environmental agents, many differences that affect health are socially rather than biologically mediated. Of course, the factors in Table 3 reflect only part of the picture, as a complete review of these issues is beyond the scope of this article. Health status and the presence or absence of diseases and disorders noted here, including nutrition, smoking, physical activity and obesity, all affect human biology, but the causes of these conditions are not solely biological and are also socially mediated. Race is not specifically listed in Table 3 because of the combination of biological and social aspects of race. While race can be seen as having a genetic component of heritable physical traits, the social constructs of race in a modern society are recognized as determining many of the stress-related factors we discuss here.

## Community-Level Analysis of Human Vulnerability

The association between specific community characteristics and exposure to environmental hazards has not been studied to identify vulnerability to cumulative risk. Given this paucity of scientific evidence, our focus is on understanding potentially relevant contextual characteristics, the plausibility of associations with health outcomes, and the delineation of a research agenda to explore these relationships.

When associations have been identified between community characteristics and health, a major challenge has been in distinguishing selection effects from causal effects. Multilevel analyses have found that the overall social and economic characteristics of residential areas are associated with a broad range of health outcomes independent of individual indicators of SES (Pickett and Pearl 2001). Diez-Roux et al. (2001) found that persons residing in disadvantaged neighborhoods in general had a higher incidence of heart disease than persons living in more advantaged neighborhoods, even after adjustment for risk factors and a broad range of personal factors.

### Residential segregation

Residential segregation by economic status and especially by race is a major characteristic that can shape differential exposure to environmental risks (Morello-Frosch and Lopez 2006). The racial/ ethnic and socioeconomic composition of communities predict a broad range of characteristics including housing, transportation, school, occupational structure, and more (Massey and Denton 1993; Williams and Collins 2001). Segregation shapes all institutions in geographically segregated areas, undermining the quality of schools, homes, transportation, commercial facilities, and safety and security (Earls and Carlson 2001). Only two studies have specifically examined links between segregation and environmental health and found that communities residing in segregated metro areas also bear a disproportionate burden of cancer risks associated with ambient air toxics (Lopez 2002; Morello-Frosch and Jesdale 2006).

Several studies have related the level of segregation to rates of morbidity and mortality, showing that residential segregation is related to elevated risk of cause-specific and overall adult mortality (Collins and Williams 1999; Fang et al. 1998; Guest et al. 1998; Polednak 1993), infant mortality (LaVeist 1989, 1992, 1993; Polednak 1991) and tuberculosis (Acevedo-Garcia 2001). At the same time, one study found that residential segregation was unrelated to infant mortality rates (Polednak 1996).

A growing body of research also suggests that communities characterized by racial and economic segregation are disproportionately exposed to a broad range of environmental hazards. Hazardous waste facilities are disproportionately located in poor and minority neighborhoods (e.g., Bullard 1983; Commission for Racial Justice 1987; Mohai and Bryant 1992; Pastor et al. 2001). These communities are also more likely to be exposed to a broad range of air contaminants because of poor outdoor and indoor air quality (Sexton et al. 1993). Other evidence suggests that these communities are also differentially exposed to pesticides and lead (Moses et al. 1993) and contaminated water (Calderon et al. 1993).

### Social capital

Social capital has emerged as a multifactorial resilience resource that can enhance health and buffer the negative impact of exposure to a variety of stressors. The term is used to capture community capacity and empowerment with an emphasis on social networks, trust, and political participation (Earls and Carlson 2001). Individuals and communities can use social capital to build resources (including health) and to address social problems. Several studies indicate that social capital is related to a broad range of health outcomes and violence (Lochner et al. 2003; Sampson et al. 1997). At the same time, several critiques of the construct (Labonte 1999; Leeder and Dominello 1999; Morrow 1999) have argued that current operationalizations of social capital are “deficient in theoretical coherence” (Earls and Carlson 2001).

Although scant research is available on the association between social capital and vulnerability or resilience in the face of environmental hazards, Rich et al. (1995) have outlined a comprehensive model through which processes of community empowerment can be mobilized in the face of local environmental hazards. Using a case study of community opposition to a sludge spreading facility in New York, the authors describe the disempowering potential of local environmental hazards and show how a partnership approach to community decision making can minimize the negative impact of environmental hazards in the life of the community. They found a great range of key contextual variables can determine a community’s capacity to respond.

### Community contexts can affect health in multiple ways

Community contexts can determine the level of the exposure to environmental and psychosocial risks (Gee and Payne-Sturges 2004; Jerrett and Finkelstein 2005; Morello-Frosch and Lopez 2006). Many predictors of health status that are typically measured at the individual level are also influenced by larger residential and occupational contexts. Nutritional status (Morland et al. 2002) and obesity (Ellaway et al. 1997), reduced physical activity levels (Shenassa et al. 2006), and cigarette smoking (Miles 2006) are influenced by community characteristics even after accounting for individual socioeconomic and demographic factors (Hillemeier et al. 2003). Long-term exposure to disadvantaged contexts can lead to altered physiologic profiles that can increase susceptibility to a broad range of environmental exposures (Geronimus 1986; 1992; Rich-Edwards 2003). Finally, characteristics of the social context can interact with individual risks and resources to increase either vulnerability or resilience.

### Conceptual model for vulnerability in cumulative risk assessment

**Figure 1:**
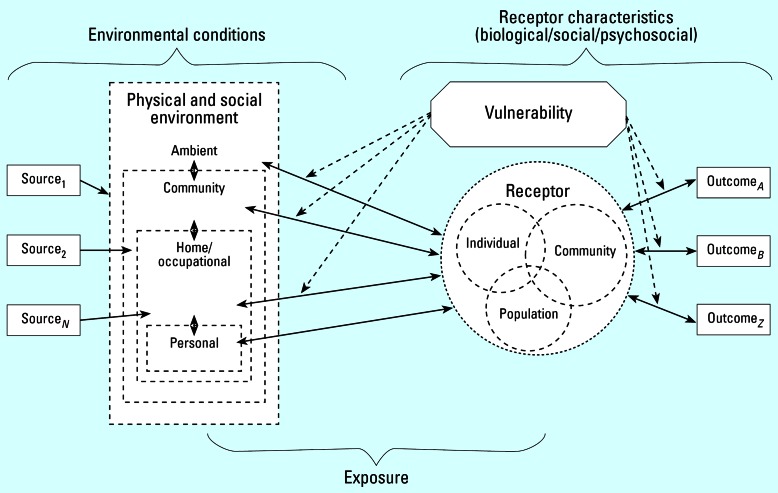
Conceptual model for considering vulnerability in cumulative risk assessment. The risk paradigm is depicted in a left-to-right flow with sources of stress on the left, exposure pathways to receptors in the center, and outcomes on the right. The receptors—individuals and groups—are shown as circles. Vulnerability factors can act at the level of how stressors interact with the receptor (left of receptors), or how receptors respond to the stress (right of receptors).

A conceptual model for how vulnerability may be incorporated into cumulative risk assessment is depicted in Figure 1. Two examples are presented briefly in Supplemental Material, Appendix B (http://www.ehponline.org/docs/2007/9332/suppl.pdf), to illustrate the conceptual model for humans and for ecologic systems. The conceptual model (Figure 1) is based on a standard risk paradigm, source to response moving from left to right. This model indicates feedback, interaction, and overlap among the key components. Dashed lines around the environmental and receptor components reflect the dynamic and fluid nature of these entities. Depending on the situation, a community may be the receptor, or the community may be the environment of an individual or population-level receptor. Two-way arrows indicate the complex interactions between environment and receptor as well as the impact of an outcome on the subsequent vulnerability of a receptor. The element of temporal and spatial patterns associated with characteristics of the model components and the interaction of these is important for application of this model but is not depicted

### Comprehensively characterizing the context

Recently, Hillemeier et al. (2003) outlined 12 overarching dimensions of contextual characteristics that may affect health. These components were identified as part of a consultative process to develop a comprehensive community contextual health profile. Specific subcomponents were identified for each of the 12 dimensions. Criteria for inclusion included conceptual relevance and the availability of data at the local level. The 12 dimensions and their specific subcomponents are economic, employment, education, political, environmental, housing, medical, government, public health, psychosocial, behavioral, and transport. These dimensions and associated subcomponents include characteristics of both the environment and the receptor.

### Guidelines for studying community effects

In many studies of “community effects,” the underlying processes are not measured or even specified. There is a need for carefully articulated theoretical frameworks and processes and direct assessment of the relevant aspects of communities. Thus, beyond identifying the important aspects of “community,” an even greater challenge is to clearly understand relationships among these various factors and the role in affecting vulnerability and resilience.

Communities are embedded in larger geographic/political environments and events in a given area are affected by phenomena of the larger region just as what happens to that larger region affects the communities therein. Characteristics of adjacent geographic areas may also have consequences for exposure to risk within a given residential area, as shown for birth weight in Chicago neighborhoods (Morenoff 2003). These contextual effects on birth weight extended beyond the immediate environment to the wider geographic neighborhood region.

## Population-Level Analysis and Vulnerability

### “Population” in ecology and public health

In ecologic systems, a population is a grouping that can be described either in terms of ecologic function or degree of reproductive interaction. In ecologic sciences a population is typically the unit of study and the entity to be preserved. In public health, population does not have a functional definition in the same way. The health sciences seek to understand and advance health as a group phenomenon, at the level of the “population.” The term population is used to mean a variety of types of groups in public health such as age, sex, occupation, social status, education, etc. In epidemiology, a population is something that can be defined by criteria used in a study design. How populations are defined is related to a significant extent to study design.

### The “vulnerability” of human populations

Within the context of this analysis, we are using three levels to consider attributes that describe vulnerability: the individual, community, and population. There are two reasons that consideration at the population level may be warranted for vulnerability and cumulative risk assessment. One is that the analysis is intended to support development of tools for analysis that will ultimately support policy change and intervention. The other, discussed below, is that many of the stressors significant for this overall assessment may affect individuals but be amenable to mitigation at other levels.

Examples of individual environmental characteristics cited in this analysis (Tables 2 and 3), such as poor neighborhood quality, substandard housing, job stress, occupational noise exposure, low SES, and higher cumulative doses of exposure, can be examined at multiple levels. Many of these factors could be addressed for individuals by actions focused on individuals, particularly those that would remove the individual from the environment of concern. But it is also possible, and sometimes desirable, to solve them at the community level through social policies to reduce risks (NEJAC 2004).

Individual vulnerability factors identified in Table 2 also include several that might be addressed at the individual, community, or population level. Poor nutrition, smoking, substance abuse, obesity, and lack of physical activity can all be seen to be either individual issues or problems or as social issues or problems that are amenable to being addressed through larger actions. Nutrition is a good example. Some analyses consider poor nutrition to be a “lifestyle” factor under the control of each individual. However, more progressive public health approaches would see nutrition as also being influenced by such social factors as availability of good quality and affordable food and disparities in access to grocery stores. These are significant concerns in many poor minority communities.

### Ecologic communities

An ecologic community is broadly defined as a group of plant and animal species interacting in a given place and time (Krebs 1985). These interactions are generally complex and involve factors such as habitat and climate as well. Because of the complexity of these interactions, predictions about a community’s differential preparedness and ability to respond can be difficult. Typical measures of community condition are as follows:

- Species diversity—the variety of species living within an area.
- Species richness—the number of species in a community, regardless of phylum.
- Abundance—the number of individual specimens of a species.
- Niche—the particular role played by a particular species.
- Total biomass—the weight of all the organisms.
- Variance—in any metric used above.

These measures are all linked together, often reciprocally, with higher diversity associated with greater number of niches within the community (Shmida and Wilson 1985). However, these metrics are not always linked to a community’s ability to withstand or resist change. Species diversity has long been hypothesized to be one of the primary indicators of ecosystem health and stability (Elton 1958; Goodman 1975; MacArthur 1955; Pimentel 1961), but the scientific community is not in full agreement on this point (Kimmerer 1984; Pimm 1979, 1980). To fully evaluate the state of a community’s vulnerability, the full structure of the community should be determined along with the identification of those species performing vital ecologic roles.

Table 4 is a list of vulnerability factors pri-oritized by the factors and characteristics influencing vulnerability on the basis of the data compiled in this article. Two case studies examining individual and community risk factors are provided in Supplemental Material, Appendix B (http://www.ehponline.org/docs/2007/9332/suppl.pdf). Lists and methodologies for measuring levels of vulnerability are given in Supplemental Material, Appendix C (http://www.ehponline.org/docs/2007/9332/suppl.pdf), and can be used in conjunction with the rankings in this list to develop specific methods.

Two types of factors, environmental and receptor, contribute to vulnerability. These are listed in Table 4 by rank according to the qualitative evaluation by the present authors.

## Recommendations and Next Steps

Our principal recommendation is to focus resources on understanding and eventually changing those conditions and characteristics of communities that increase vulnerability. These efforts must not be misdirected to focus on personal, community and population factors that improve resilience. Rather, the focus must remain on preventing the causes of vulnerability. Such an effort is needed for at least three reasons. One, research with children on cumulative risk and protective factors shows quite clearly that the impacts of cumulative risk exposure far outweigh the mitigating effects offered by protective factors [see Sameroff et al. (1998) studies on children’s IQ]. Two, a focus on resilience may redirect attention to the subset of individuals capable of withstanding cumulative risks rather than efforts to improve environmental quality. Three, emphasis on receptor characteristics that moderate environmental risk impacts can all too easily lead to blaming the victims of poor environmental exposure rather than fundamentally improving community capacity and well-being (Earls and Carlson 2001).

It is important to maintain our focus on the environmental causes of ill health effects as we study them within a more realistic ecologic context. Toward that end, we believe the following steps would greatly improve our ability to address cumulative risk:

- Develop a formula/method using quantifiable metrics to estimate vulnerability for human populations and communities.
- Investigate the effectiveness of any formula or method in predicting vulnerability using cases such as hurricanes Katrina and Stan and the tsunami of December 2004 (Allenby and Fink 2005).
- Develop a method or formula using ecologic metrics to estimate vulnerability for ecologic units. It may be necessary to develop different formulas for different types of systems, such as terrestrial versus aquatic, Arctic, deserts, etc.
- Perform studies to verify effectiveness of these ecologic metrics in assessing vulnerability
- Integrate quantified levels of vulnerability into cumulative risk framework.

Currently, the U.S. EPA purports to protect human health at the individual level and wildlife at the population level, with the exception of endangered species. This approach omits the community level of organization that we recommend for use in cumulative risk assessment. Acting on these recommendations would have a significant effect on policy and will, therefore, require attention at the upper management levels. The U.S. EPA and other federal agencies should undertake both short-and long-term efforts to incorporate vulnerability into risk assessment, especially cumulative risk assessments. In the short run, important vulnerability factors can and should be incorporated into current risk assessment practices. To accomplish this goal, the U.S. EPA needs to fund in-house training and educational activities for the U.S. EPA professional staff to increase awareness and understanding of cumulative environmental risk and vulnerability issues. In the long run, research is needed to develop ways to measure the known vulnerability factors and incorporate these into risk practices. Research is also needed to understand and identify vulnerability in both human and ecologic risk situations (Allenby and Fink 2005). We have highlighted those factors that researchers found in the course of other studies, but few if any research efforts have intentionally sought the factors that increase vulnerability. The next level of research in the area of vulnerability for cumulative risk needs to be intentional.

## Figures and Tables

**Table 1:** Examples of specific vulnerability factors.

Environmental conditions (habitat quality)	Receptor characteristics (individual or group quality)
Location	Biological factors
Geographic area	Genetics
Urban	Gender
Rural	Genetic diversity
Proximity to industrial sites	Genetic flux
Proximity to roads and traffic	Susceptibility
Time indoors, time outdoors	Developmental or life stage
Quality of setting	Age
Natural environment	Population structure
Air quality	Physical health status
Water quality	Low birth weight
Climate, habitat	Chronic disease-obesity
Built environment	Compromised immune function
Land use	Asthma
Housing quality	Acute disease-exposure
Housing density	Infection
Occupant density	Nutrition
Sanitation	Injury
Traffic density	Psychologic factors
Noise	Mental/emotional health
Social environment	Depression
Segregation	Hostility
Crime	Poor coping skills
Chaos	Temperament
Conflict	Adaptability
Social support	Intensity
Immigration/emigration	Mood
Family or group stability	Persistence/attention span
Violence	Distractibility
Racism	Sensitivity
Resources	Activities/behaviors
Social capital	Physical activity
Wealth	Hygiene
Employment opportunities	Diet
Schools	Product use
Medical care	Smoking
Food availability	Substance abuse
System complexity and redundancy	Religious practice
	Social factors
	Race/ethnicity
	SES
	Population size
	Diversity
	Number of species
	Other
	Marital status
	Educational status

**Table 2:** Environmental vulnerability factors affecting individuals.

Household	Community	Institutions
Low SES	Low neighborhood quality	Poor quality schools
Family turmoil	Crime and violence	Poor quality medical care
Marital instability	Low social capital	Job strains (high demands, low control, no security)
Cold, harsh parenting	Deviant peers	Access to economic opportunities
Separation from family	Poor social support	
Poor housing quality	Noise	
Crowding	Segregation	
Chronic stressor exposure	Poverty	
Residential instability	Income inequality	
Chaotic, lack of structure, routines, rituals		

**Table 3:** Receptor vulnerability factors affecting individuals.

Biological	Personality and intelligence	Interpersonal
Gender	Negative emotionality, pessimism, difficult temperament	Poor self-regulatory skills (impulsive, attention focusing difficulties)
Genetic predispositions	Hostility and aggressiveness	Poor coping skills
Compromised immune function	Low mastery beliefs, low self-efficacy	Shyness, extreme introversion
Allergies	Depression and anxiety	
Asthma	Low intelligence	
Nutrition		
Smoking		
Substance abuse		
Low birth weight/prematurity		
Obesity, physical activity, age		

**Table 4:** Factors contributing to vulnerability.

Environment	Receptors
Household	Receptor factors
1. Low SES	1. Genetics
2. Chronic stressor exposure	2. Development of life stage
3. Family turmoil	3. Physical health status
4. Chaos—lack of structure and rituals	4. Mental/emotional health status
5. Poor housing quality	5. SES
6. Cold, harsh parenting	6. Race/ethnicity
7. Marital instability	7. Culture
8. Residential instability	8. Temperament
9. Separation from family	Individual level
10. Crowding	1. Diet/nutritional status
Community factors	2. Social support
1. Low neighborhood/housing quality	3. Psychosocial stress
2. Crime and violence	4. Low SES/poverty
3. Crowding	5. Health behaviors
4. Food supply	Personality/intelligence
5. Access to health care	1. Negative emotionality—pessimism, difficult temperament
6. Concentration of poverty	2. Depression/anxiety
7. Poor social support	3. Poor coping skills
8. Racial segregation	4. Low mastery beliefs/low self-efficacy
9. Noise	5. Poor self-regulatory skills
Institutions	6. Shyness/extreme introversion
1. Poor-quality schools	7. Hostility and aggressiveness
2. Job strain	8. Low intelligence
3. Poor-quality medical care	Biological
Physical conditions	1. Racial minority
1. Location	2. Allergies and asthma
2. Quality of setting	3. Smoking
3. Activities	4. Gender
Social conditions	5. Compromised immune function
1. Social capital	6. Low birth weight/prematurity
2. Resources	7. Obesity/low physical activity
3. Behavior	8. Substance abuse
	Other factors
	1. Habitat quality
	2. Age
	3. Population quality
	4. Health status
	5. Multiple stressors
